# The Effect of Incentives on Disaster Mitigation Behavior: An Age-Based Analysis

**DOI:** 10.1093/geroni/igaf122.816

**Published:** 2025-12-31

**Authors:** Wenqian He, Jeffrey Wickliffe, Zhen Cong

**Affiliations:** The University of Alabama at Birmingham, Birmingham, Alabama, United States; The University of Alabama at Birmingham, Birmingham, Alabama, United States; Chapman University, Orange, California, United States

## Abstract

**Background:**

As climate change accelerates, the frequency and severity of natural disasters are increasing. However, most individuals cannot get sufficient protective measures due to financial pressure or environmental barriers. This study aims to examine how different incentives influence people’s willingness to take mitigation behavior.

**Methods:**

Data were collected from 781 tornado survivors in Texas, Alabama, and Tennessee, as part of the ‘Vulnerability and Resilience to Disasters’ project. Participants were randomly assigned to 12 conditions based on cost coverage ratios (25%, 50%, 75%), improvement types (storm shelters, structural reinforcement), and incentive forms (cash rebates, insurance discounts). Multivariate logistic regression models were used, with a focus on age differences (< 49 years vs. ≥50 years).

**Results:**

Higher cost coverage increased protective willingness, and participants are more willing to get structural upgrades than storm shelters. No significant interaction effects were found between incentive methods. Besides, older participants, people with minor children, people experienced stress during the last tornado, and people anticipated future tornadoes were more likely to take mitigation behavior. Stress increased younger participants’ willingness, while the presence of minor children increased older participants’ willingness. 75% cost coverage was effective across all groups, while 50% coverage alone worked for younger participants but need to be combined with insurance discounts to work for older participants.

**Conclusion:**

Cost coverage is important in disaster mitigation. Younger participants are more likely to be driven by stress, while older participants prefer combined incentives and prioritize family safety. Policies should consider demographic differences to enhance effectiveness and equity.

